# Case report: Active clinical manifestation of endocardial fibroelastosis in adolescence in a patient with mitral and aortic obstruction–histologic presence of endothelial-to-mesenchymal transformation

**DOI:** 10.3389/fcvm.2022.1041039

**Published:** 2022-11-30

**Authors:** Daniel Diaz-Gil, Chrystalle Katte Carreon, Natalia Silva-Gomez, Alan E. Benheim, Sitaram M. Emani, Pedro J. del Nido, Gerald R. Marx, Ingeborg Friehs

**Affiliations:** ^1^Department of Cardiac Surgery, Boston Children’s Hospital, Harvard Medical School, Boston, MA, United States; ^2^Klinik für Kinderherzmedizin und Erwachsene mit angeborenen Herzfehlern, University Medical Center Hamburg-Eppendorf, Hamburg, Germany; ^3^Cardiac Registry, Department of Cardiology, Pathology, and Cardiac Surgery, Boston Children’s Hospital, Boston, MA, United States; ^4^Department of Pathology, Boston Children’s Hospital and Harvard Medical School, Boston, MA, United States; ^5^Division of Cardiology, Inova L.J. Murphy Children’s Hospital, Falls Church, VA, United States; ^6^Department of Cardiology, Boston Children’s Hospital, Harvard Medical School, Boston, MA, United States

**Keywords:** endocardial fibroelastosis, hypoplastic left heart complex, endothelial-to-mesenchymal transformation, ABL1, flow disturbance

## Abstract

This is the first description of active clinical manifestation of endocardial fibroelastosis (EFE) and remodeling of the endocardium *via* endothelial-to-mesenchymal transformation (EndMT) in an adolescent with Shone’s variant hypoplastic left heart complex (HLHC) and a genetic heterozygous ABL1 variant. While EFE has not been typically associated HLHC or Shone’s syndrome, in this patient flow alterations in the left ventricle (LV), combined with genetic alterations of intrinsic EndMT pathways led to active clinical manifestation of EFE in adolescence. This case emphasizes that new therapies for EFE might need to focus on molecular factors influenced by intrinsic and extrinsic stimuli of EndMT.

## Introduction

Hypoplastic left heart complex (HLHC) which also covers Shone’s syndrome is a rare congenital heart disease comprised of severe obstructive lesions involving the left-sided inflow and outflow tracts (1, 2) with an incidence of 154–279 per million live births (3). In Shone’s variant HLHC, the left ventricle (LV) is narrow but apex forming, the mitral valve (MV) can be stenotic due to annular hypoplasia or a parachute configuration, and the LV outflow tract (LVOT) shows multilevel obstructions. The spectrum of disease ranges from relatively mild to severe forms, and while supramitral fibroelastic membranes contributing to MV obstruction are common in this disease entity, LV endocardial fibroelastosis (EFE) has not been associated as a major factor in Shone’s variant HLHC (4).

Based on clinical observation from the LV rehabilitation approach at Boston Children’s Hospital, EFE involves the LV myocardium, contributing to LV systolic and diastolic dysfunction. Moreover, EFE is observed during surgery in multilevel obstructive lesions involving the left atrium, MV apparatus and LVOT. EFE is a thickened subendocardial layer of collagen and elastic, and occurs as early as during fetal development. Hypothetically, EFE can prevent normal growth and development of the LV but potentially might also contribute to the pathologic changes of the MV and LVOT and aortic valve. We have also recently reported that flow disturbances are associated with the remodeling of the endocardium through a process called endothelial-to-mesenchymal transformation (EndMT), which we have shown as an underlying root cause for EFE formation (5–7). EndMT is the phenotypical switch of endocardial endothelial cells to mesenchymal cells which is a normal developmental process during fetal heart morphogenesis giving rise to the septa and valves (8). Despite EFE being an early childhood disease, we present the case of an adolescent with active clinical manifestation of EFE through activation of EndMT in the entire LV cavity, MV and subaortic region at 14 years of age.

## Case presentation

This male, Caucasian patient of Armenian descent presented at 2 days of age with Shone’s variant anatomy, including MV stenosis, subaortic stenosis, a non-stenotic bicuspid aortic valve (right non-fusion), and aortic coarctation. At the referral hospital, the patient underwent a coarctectomy with end-to-end anastomosis in the newborn period, followed by several transcatheter interventions for recoarctation, resulting in coarctation recurrence, and the placement of multiple stents from the distal transverse arch to the thoracic descending aorta. He also underwent multiple catheter-based aortic balloon valvuloplasties for mild aortic valve stenosis. At 6 years of age, the patient required a surgical mitral valvuloplasty with separation of papillary muscles and resection of stenosis in the inter-chordal space in repair of a worsening mitral stenosis. Additionally, a septal myectomy was performed to relieve a muscular subaortic stenosis. After surgery, the patient had an uneventful recovery and returned to full activities with good stamina.

Whole-exome sequencing ruled out an underlying aortopathy (suggested due to the unusual aortic disease with extensive stenosis of the thoracic aorta requiring multiple stent placements), but demonstrated a heterozygous ABL1 variant (p.Pro329Arg (CCG > CGG): c.986 C > G in exon 6 on the ABL1 gene (NM_007313.2), ACMG/AMP classification as variant of uncertain significance). *In silico* analysis performed during whole exome sequencing supported that this missense variant has a deleterious effect on protein structure and function of ABL1. In addition, the patient’s past medical history was significant for preterm birth, initially delayed milestones, short stature, dysmorphic facial features, cryptorchidism, webbed short fingers, narrow tapered palpebral fissures,– all features previously described in patients with similar heterozygous germline ABL1 variants, summarized as ABL1-associated congenital heart defects and skeletal malformation syndrome (9–11). Of note, the mutation was inherited from the father, who has no history of congenital heart disease, suggesting variability in penetrance.

**FIGURE 1:**
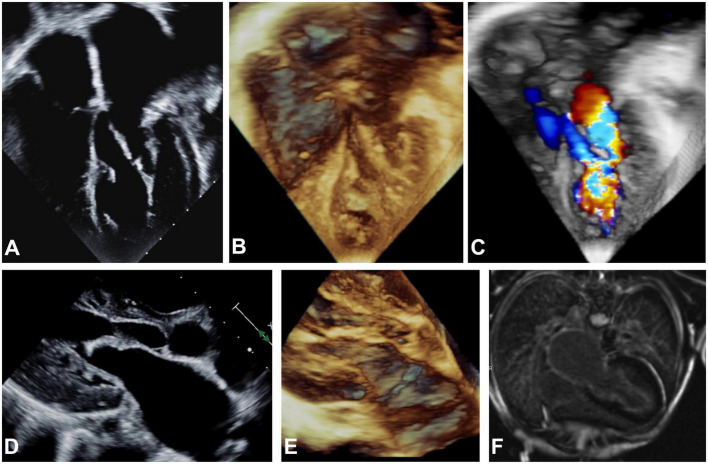
Echocardiographic apical 4-chamber view **(A–C)**, parasternal long axis **(D,E)** and MRI **(F)** imaging before EFE resection. 2D **(A,D)** and 3D **(B,C,E)** echocardiography showing hyperechogenic thickening of the endocardium on atrial, mitral valve, LV and LVOT level consistent with generalized EFE in the left sided structures of the heart is shown. Severe dilation of the left atrium can be noted. **(C)** Severe turbulences are detected by color Doppler in all left sided structures affected by EFE as compared to physiological color Doppler appearance in the right sided structures of the heart that are not affected by EFE. **(F)** Late gadolinium enhancement on endocardial level encapsulating all structures of the left atrium and ventricle is depicted.

At 13 years old, the patient presented with an asymptomatic aortic valve gradient of 55 mmHg, which was reduced to 30 mmHg *via* balloon valvuloplasty. The pulmonary capillary wedge pressure was elevated to 21 mm Hg with a mitral stenosis mean gradient of 9 mm Hg. In the following year, the patient reported a steady decrease in energy levels, accompanied by leg numbness, and limited stamina even after short periods of rest. The patient was referred to our institution, and echocardiographic and MRI imaging revealed global LV hypertrophy and dysfunction. Thickened amorphic tissue with bright signals from the base to LV apex consistent with a diagnosis of EFE were detected on the endocardial LV surface involving the MV and subaortic region. Marked thickening of the atrial surfaces of the anterior and posterior MV leaflets in the mid leaflet region prevented effective leaflet coaptation (Figure 1). There was LVOTO (peak gradient if 45 mmHg, mean gradient of 22 mmHg), from the subaortic region extending to the undersurface of the bicuspid aortic leaflets. At repeat catheterization, LV filling pressures were increased to 20 mmHg. On cardiac CT, severe in-stent stenosis along nearly the entire length of the stented descending aorta was seen. Overall, the patient’s symptoms were attributed to an inability to significantly increase his cardiac output due to inflow and outflow obstruction and decreased diastolic compliance due to EFE.

The patient underwent surgery consisting of a mitral valvuloplasty with surgical resection of the tethering attachments to the anterior and posterior leaflets, thinning of the leaflets with the removal of thickened amorphous tissue (presumed to be EFE), mobilization of the leaflets, and the anterior papillary muscle. Furthermore, tissue was resected from the entire LV, the subaortic region, and the aortic valve, accompanied by valvuloplasty and commissurotomy.

**FIGURE 2:**
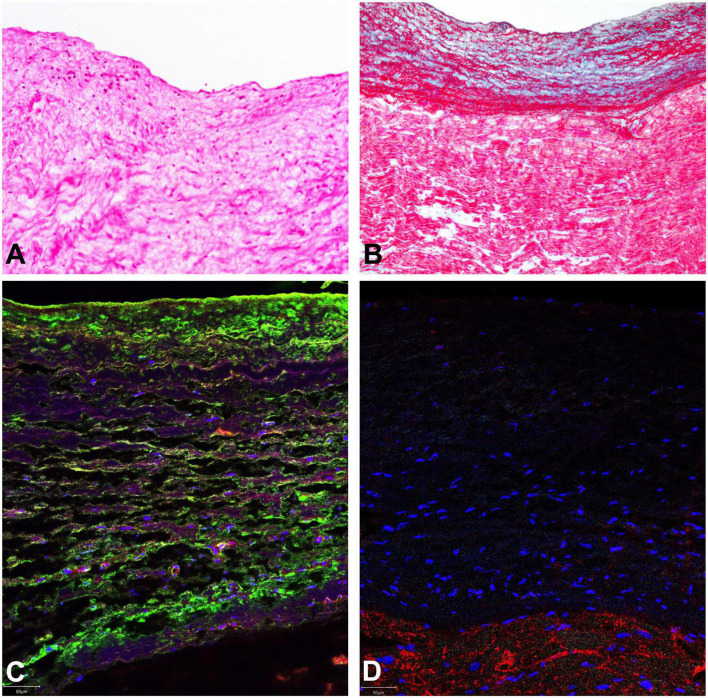
Representative histological evaluation of resected EFE tissue. Organized structured avascular collagen and elastin matrix with scarce cellularity, consistent with the histological picture of EFE is shown **(A)** H&E staining shows only sparse mononuclear likely mesenchymal stromal cells **(B)**. Masson’s trichome stain displays a thickened subendocardium with multiple and often alternating layers of elastic fibers (red wavy profiles) and fibrous tissue (blue) with extension into the subjacent myocardium. **(C)** Double-staining of endocardial endothelial cells with VE-cadherin (red) and alpha-smooth muscle actin (green) were performed to detect EndMT. A representative section is shown. The majority of endocardial endothelial cells express both markers at the same time, indicating active EndMT. **(D)** Lack of predominant inflammatory invasion of EFE tissue is confirmed by negative immunofluorescence staining for CD45 (absence of green). Cardiomyocytes are counterstained with Troponin I (red).

All resected tissues were comprised of paucicellular organized avascular collagen and elastin matrix, which is consistent with the histological picture of EFE (5, 6). Furthermore, pathological evaluation of the subaortic obstruction revealed fragments of endocardium-lined fibromyxoid tissue with scant attached myocardium. Additionally, immunofluorescence analysis of the resected EFE tissue from all areas showed a high number of cells expressing both endothelial and mesenchymal markers, indicative of active EndMT (Figure 2).

The immediate post-operative course was uneventful with only mild residual mitral stenosis (Doppler echo mean gradient 3–4 mmHg) and a residual gradient over the aortic valve (peak gradient 33 mmHg, mean gradient of 21 mmHg). At 3 months follow-up, B-type natriuretic peptide had normalized to 91 pg/ml from 827 pg/ml preoperatively. At 6 months follow-up, echocardiography showed a remaining mild LV hypertrophy with good systolic function, and mild valvular mitral and aortic stenosis with a mean gradient of 5–6 and 21 mmHg, respectively. A marked improvement in diastolic flow parameters was noted. The patient was doing well clinically without any apparent signs of congestive heart failure on his standard heart medication regimen. He now can exercise without limitations.

## Discussion

This is the first description of active clinical manifestation of EFE and remodeling of the endocardium *via* EndMT in adolescence in an individual with longstanding congenital heart disease involving the MV, the subaortic region and the entire LV endocardium, and no previous clinical evidence of EFE. EFE was visible immediately preoperatively on echocardiography and MRI, and confirmed by histological analysis of the resected tissue, showing active EndMT. This is a signature case, since it has been postulated that EFE manifests during the fetal stage and early childhood. In this case we have histological evidence of active EFE development and associated clinical manifestation in adolescence (4). While it cannot be ruled out that some level of EFE was already present earlier in the disease course, this patient presented with marked changes in clinical symptoms and LV function, which suggested an acute clinical manifestation reflecting either *de novo* development of acute progression in adolescence.

Endothelial-to-mesenchymal transformation can be induced by different stimuli including mechanical forces or inflammation (12). As we have previously shown hemodynamic alterations, primarily flow disturbances are directly associated with the induction of EndMT (5–7). Flow disturbances had been present in this patient since birth. To rule out inflammation as a potential stimulus for EndMT, we performed histological analysis of EFE tissue. As indicated in Figures 2A,D, there was no evidence of systemic infiltration of inflammatory cells which was supported by preoperative normal total and differential leukocyte counts.

In this case, we postulate that activation of EFE manifested in the context of disturbed LV inflow and outflow. Varying degrees of altered flow patterns through the MV in combination with outflow tract obstructions have been suggested to contribute to the spectrum of critical AS to HLHS (AS/MS), with numerous studies showing that disturbed inflow through the MV is associated with the development of EFE (5, 7, 13, 14). Specifically, Sharland et al. (13) and Allan et al. (15) showed in the fetus that the initial intrauterine echocardiographic evaluation often displayed a pattern of reduced MV inflow while forward flow in the ascending aorta remained normal. Later in gestation when no antegrade flow was detected–through the MV or the aortic valve–a bright endocardial layer consistent with EFE was observed. These findings suggest that inflow disturbances caused by either MV abnormalities or increased LV filling pressures, are the underlying hemodynamic factors that are associated with EFE formation. Furthermore, the severity of the overall flow reduction and timespoints of such “insults” may play a role in ultimately developing into HLHS or–critical AS (16–18). Interestingly, in the presented patient, aortic and MV abnormalities and aortic coarctation were evident since birth. Reduction of recurrent aortic outflow tract gradients left a hemodynamic situation of MV stenosis with disturbed MV inflow and active clinical manifestation of EFE.

While the MV circumferences in patients with HLHS (AS/MS) or critical aortic stenosis may be just above or below the lower ranges of normal values, they are frequently dysplastic and stenotic, probably because of intrinsic alterations in EndMT regulation (19). In contrast, even though aortic valves are often small, they are also often formed normally (19). Furthermore, while no genetic abnormality is specific to HLHS or critical aortic stenosis, there is strong evidence supporting a genetic etiology, and certain implicated specific gene mutations like NOTCH1 play a crucial role in modulating EndMT (20–22). Not only was this patient’s predominant intracardiac defect at birth a dysplasia of the MV but he was also diagnosed with a heterozygous germline missense ABL1 variant, which while being of uncertain significance, *in silico* analysis suggested has a deleterious effect on protein structure and function of ABL1. Although the protooncogene ABL1 is well known for being part of the fusion gene BCR*ABL1 in leukemic cells (23), inherited germline heterozygous mutations in ABL1 have only recently been found to cause congenital heart defects and skeletal malformations through an increase in ABL1 kinase activity (9–11). ABL1 kinase activity influences EndMT in the entire body through inactivation of the phosphatase and tensin homolog (PTEN) (24), a mechanism that has also been implicated in LV hypertrophy and non-compaction in response to biomechanical stress (25, 26). The combination of two hits—an intrinsic EndMT alteration through his underlying genetic mutation and distinct flow disturbances in adolescence potentially led to EFE formation, and we speculate that a similar two-hit hypothesis could also be the underlying mechanism behind the development and progression of EFE in patients with HLHS (AS/MS)/critical aortic stenosis spectrum disease.

In conclusion, this case emphasizes the likely multifactorial nature in development and persistence of EFE into adolescence. In this patient, presenting with distinct flow alterations in the LV, resembling well-described intrauterine patterns in patients with LV disease in the HLHS (AS/MS)/critical AS and Shone’s spectrum, in combination with genetic alterations of intrinsic EndMT pathways, led to active clinical manifestation of EFE in adolescence. A deeper understanding of genetic and transcriptional factors influenced by intrinsic and extrinsic stimuli of EndMT might lead to new therapeutic strategies (e.g., through the usage of readily available pharmacotherapies like tyrosine kinase inhibitors) for the prevention of EFE and associated congenital heart defects.

## Data availability statement

The raw data supporting the conclusions of this article will be made available by the authors, without undue reservation.

## Ethics statement

Ethical review and approval was not required for the study on human participants in accordance with the local legislation and institutional requirements. Written informed consent to participate in this study was provided by the participants’ legal guardian/next of kin. Written informed consent was obtained from the minor(s)’ legal guardian/next of kin for the publication of any potentially identifiable images or data included in this article.

## Author contributions

DD-G, GM, and IF were involved in the conceptualized of this case report. DD-G and IF designed the methodology, conducted the experiments, and wrote—original draft. DD-G and CC performed the experiments. DD-G, GM, PN, AB, and IF were responsible for clinical data acquisition. DD-G, CC, and IF performed the formal analyses. DD-G, CC, NS-G, AB, SE, PN, GM, and IF reviewed and edited the final draft. IF was responsible for the acquisition of funding. All authors contributed to the article and approved the submitted version.

## Abbreviations

EFE: endocardial fibroelastosis
EndMT: endothelial-to-mesenchymal transformation
MV: mitral valve
LV: left ventricle
LVOT: left ventricular outflow tract
PCWP: pulmonary capillary wedge pressure
HLHS: hypoplastic left heart syndrome
HLHC: hypoplastic left heart complex.
